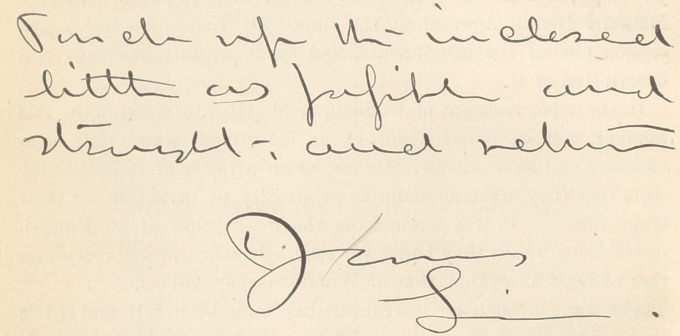# The Pot *vs.* the Kettle

**Published:** 1884-02

**Authors:** 


### THE POT *versus* THE KETTLE.

Editor Barrett has a type-writer. We know this because we saw it in his office. He uses it, sometimes, whereat every one who has been favored with a letter from our neighbor should rejoice—not, however, because of the letter as much as the comforting assurance that in his attempt to decipher it he is not imperiling his immortal soul. The purchase of the type-writer is directly due to the appeal of one sorely tried. Like the good man that he is, he went right off and bought one.—*Odontographic Journal.*

Just to show how black is the bottom of this reproaching pot, we present a *fac simile* of the chirography—if such we may call it—of the editor of the *Odontographic.* Just look at the signature. It is a *Line*, sure enough, but not a straight line, although approaching it as nearly as the indirectness of the signer would permit.

There is a French-man within a stone's throw of the publication office of the *Odontographic*, who avers that his first infraction of —we have forgotten whether it was the third or seventh commandment of the Decalogue, perhaps it was both—was directly due to an attempt to decipher some of the sprawling, back-handed, illegible hieroglyphics of Editor Line.

## Figures and Tables

**Figure f1:**